# Tinea capitis in Hainan: a prospective study

**DOI:** 10.3389/fcimb.2025.1590315

**Published:** 2025-06-23

**Authors:** Wenai Zheng, Feng Qiao, Xianxu Yang, Xiaozheng Tang, Changyan Lin, Qin Chen, Zhaobing Pan, Rongqiang Chen

**Affiliations:** ^1^ Department of Clinical Laboratory, The Fifth People’s Hospital of Hainan Province, Haikou, China; ^2^ Department of Pathology, The Fifth People’s Hospital of Hainan Province, Haikou, China; ^3^ Department of Dermatology, The Fifth People’s Hospital of Hainan Province, Haikou, China; ^4^ Department of Xiuying Outpatient, The Fifth People’s Hospital of Hainan Province, Haikou, China

**Keywords:** tinea capitis, prospective, microsporum canis, epidemiology, pathogenic fungi

## Abstract

**Objective:**

Tinea capitis, a chronic inflammatory disease of the scalp and hair shafts caused by dermatophyte infections, ​manifests clinically​ as erythematous lesions, scaling, alopecia, and pustule formation. Hainan, a tropical island province in China, exhibits distinctive climatic conditions and demographic contact patterns that ​could shape​ its pathogen spectrum. However, comprehensive epidemiological data ​remain scarce. This study ​analyzes​ the epidemiological characteristics and pathogen spectrum of tinea capitis in Hainan, China.

**Methods:**

This study ​was undertaken​ across 11 coastal and inland dermatological centers in Hainan Province (January 2023 to December 2024). A total of 76 tinea capitis patients ​diagnosed​ via mycological examination (fungal fluorescence microscopy, fungal culture with species identification) ​and corroborated​ by dermatoscopic evaluation ​were consecutively enrolled. Data on demographic characteristics, exposure history, and clinical manifestations ​were systematically collated​ using standardized case report forms. Pathogen subtyping ​was performed via​ integrated morphological identification ​supplemented by​ molecular biological analysis of the ITS region.

**Results:**

Among 76 enrolled patients, minors (≤15 years) ​accounted for​ 80.26% (61/76), with ≤10-year-olds ​representing​ 91.8% (56/61) of pediatric cases. The overall male-to-female ratio ​was recorded as​ 1:1.05 (37 males vs. 39 females), while pediatric patients (≤15 years) ​exhibited​ a 1.07:1 ratio (31 males vs. 29 females). Animal contact history ​was reported in​ 31 cases (40.79%), scalp trauma in 3 cases (3.95%), and co-occurring superficial fungal infections in 12 cases (15.79%). ​Fungal elements were detected​ via direct microscopic examination in 64 cases (84.21%). Fungal cultures ​obtained​ 45 positive isolates (19 strains in 2023, 26 in 2024), with kerion (inflammatory tinea capitis) ​exhibiting​ the highest culture positivity rate, followed by tinea alba. ​The primary pathogens identified​ were zoophilic *Microsporum canis (M*. *canis*) (20 cases, 43.79%) and anthropophilic *Trichophyton rubrum (T. rubrum*) (9 strains, 19.57%). Clinical manifestations ​comprised​ kerion (44 cases, 57.89%), tinea alba (27 cases, 35.53%), and black dot tinea (5 cases, 6.58%). Kerion cases ​were predominantly linked to​ *M. canis and T. mentagrophytes* (interdigital subtype), whereas tinea alba ​demonstrated​ infections by *M. canis and T. rubrum*. Black dot lesions ​were additionally observed​ in *M. canis* infections.

**Conclusion:**

The primary affected group is children ≤10 years old, with the most common pathogenic fungus being zoophilic *M. canis*, and clinical classification is predominantly kerion. Dermatologists should pay attention to different transmission routes and pathogen spectra.

## 1 Introduction

Tinea capitis, a chronic dermatophytic infection of the hair and scalp, causes erythema, scaling, alopecia, abscesses, and scarring after lesion resolution. This illness is the most frequent superficial fungal infection in school-aged children worldwide, with higher rates reported in resource-limited settings (Ferguson and Fuller, 2017; Rodríguez-Cerdeira et al., 2021).

The epidemiological distribution of tinea capitis pathogens exhibits regional and temporal heterogeneity, shaped by socioeconomic factors, lifestyle changes, migration trends, and host demographic dynamics. In China, rapid socioeconomic development and lifestyle changes since the 1980s have triggered a transition in predominant pathogens. Before 1985, anthropophilic dermatophytes prevailed; however, after 1985, zoophilic species—particularly M. canis —have predominated, representing >80% of cases (Zhan et al., 2015). Anthropophilic strains such as Trichophyton violaceum remain endemic solely in localized areas of southeastern and northwestern China. The dominant clinical manifestations of tinea capitis in China have shifted chronologically: favus (1950s) was superseded by white tinea (peak prevalence in the 1990s), with black dot tinea emerging as the most prevalent form in the 21st century (Wan and Wu, 2022). Comparable epidemiological trends are observed globally (Rodríguez-Cerdeira et al., 2021). *M. canis* continues to be the predominant zoophilic pathogen internationally, whereas Trichophyton violaceum and Trichophyton tonsurans are the most epidemiologically significant anthropophilic species.

This study ​utilized​ a prospective, descriptive design ​to prospectively collate data from​ tinea capitis cases diagnosed across ​multi-city​ dermatology departments, specialized hair/scalp clinics, and dermatology prevention/treatment centers in Hainan Province, China (January 2023–December 2024). The investigation ​systematically examined​ the age and gender distribution, risk factors, and pathogen spectrum of tinea capitis in Hainan, ​with the objective of establishing​ an evidence-based foundation ​to advance understanding of​ its epidemiology, diagnosis, and clinical management in the region.

## 2 Material and methods

### 2.1 Clinical data

A total of 76 cases of tinea capitis diagnosed via clinical fungal microscopy and culture were included in the study from January 2023 to December 2024. All subjects received written informed consent. The study was conducted under the Declaration of Helsinki and approved by the ethical standards of the Ethics committee of the Fifth People’s Hospital of Hainan Province (IRB no. 2020-020). Records were kept of demographic features, types of tinea capitis, and species of pathogenic fungi, along with skin dermoscopy findings.

### 2.2 Epidemiological information collection

An epidemiological survey form was filled out with informed consent from patients and their families. The survey form included key information such as age, gender, long-term residence, presence of other diseases, history of animal contact, type of animals contacted, presence of concurrent superficial mycosis, and type of concurrent superficial mycosis among cohabiting and close contacts.

### 2.3 Fungal fluorescence microscopy

Alcohol (75%) was used to disinfect the affected area, and scales, pus, or broken hairs were collected using a scalpel or forceps. These samples were placed on a slide and directly stained with fungal fluorescence dye (Beckman Coulter). Under a fluorescence microscope, the following fungal microscopy findings were recorded: intramural hyphae, intramural spores, intramural hyphae and spores, extramural hyphae, extramural spores, and extramural hyphae and spores. Multiple types could be present in the same patient’s sample. If none of these were observed, scalp hyphae were recorded.

### 2.4 Fungal culture and identification

Remaining specimens were inoculated onto Sabouraud dextrose agar (SDA) and incubated at 25-28°C for 14 days. Fungi were preliminarily identified based on colony growth rate, pigmentation, size, conidia morphology, and hyphal characteristics. For rare or difficult-to-identify species, further small-scale cultivation and morphological analysis were performed.

### 2.5 Molecular biology identification

*M*. *canis* was identified by morphology, while the internal transcribed spacer (ITS) regions of the other species were amplified and sequenced, (ITS1 5’-TCCGTAGGTGAACCTGCGG-3’; ITS4 5’-TCCTCCGCTTATTGATATGC-3’) (Chen et al., 2022).

All isolates were subcultured on potato dextrose agar (PDA) for 7 days before sequencing. PCR amplification was performed using universal fungal primers ITS1 and ITS4, followed by sequencing of the amplified products. Reference sequences with high similarity to known sequences were downloaded from BLAST results and compared with rDNA-ITS sequences in the GenBank database to assist in strain identification.

### 2.6 Statistical analysis

Descriptive statistics were used to analyze the demographic and clinical data of patients diagnosed with tinea capitis, including ​sex, age groups, clinical subtypes (e.g., kerion, tinea alba), and pathogen groups. Categorical variables (e.g., ​sex, ​pathogen species, exposure history) were compared across groups ​using Fisher’s exact test​ (employed for small sample sizes as an alternative to the chi-square test), ​with statistical significance defined as a two-tailed​ p ​​< 0.05. All statistical analyses were performed using SPSS version 23.0 software (IBM, Armonk, NY, USA).

## 3 Results

### 3.1 Epidemiological characteristics

From January 2023 to December 2024, 76 cases of tinea capitis were included, with 37 males and 39 females (male-to-female ratio = 1:1.05). The majority of patients (80.26%, 61/76) were minors (≤15 years old), with 91.8% (56/61) being ≤10 years old. The male-to-female ratio among minors was 1.07:1 (31:29). Thirty-one patients (40.79%) had a history of animal contact, mainly involving cats and dogs (18 and 11 cases, respectively), with 28 children under 10 years old (90.32%) and 3 adults (9.68%). Three patients (3.95%) had a history of scalp trauma, and 12 patients (15.79%) had concurrent superficial mycosis at other sites. Most patients (64.46%, 49/76) were from Haikou City and surrounding areas (**Supplementary Table 1**).

### 3.2 Clinical diagnosis and classification

**Figure 1:**
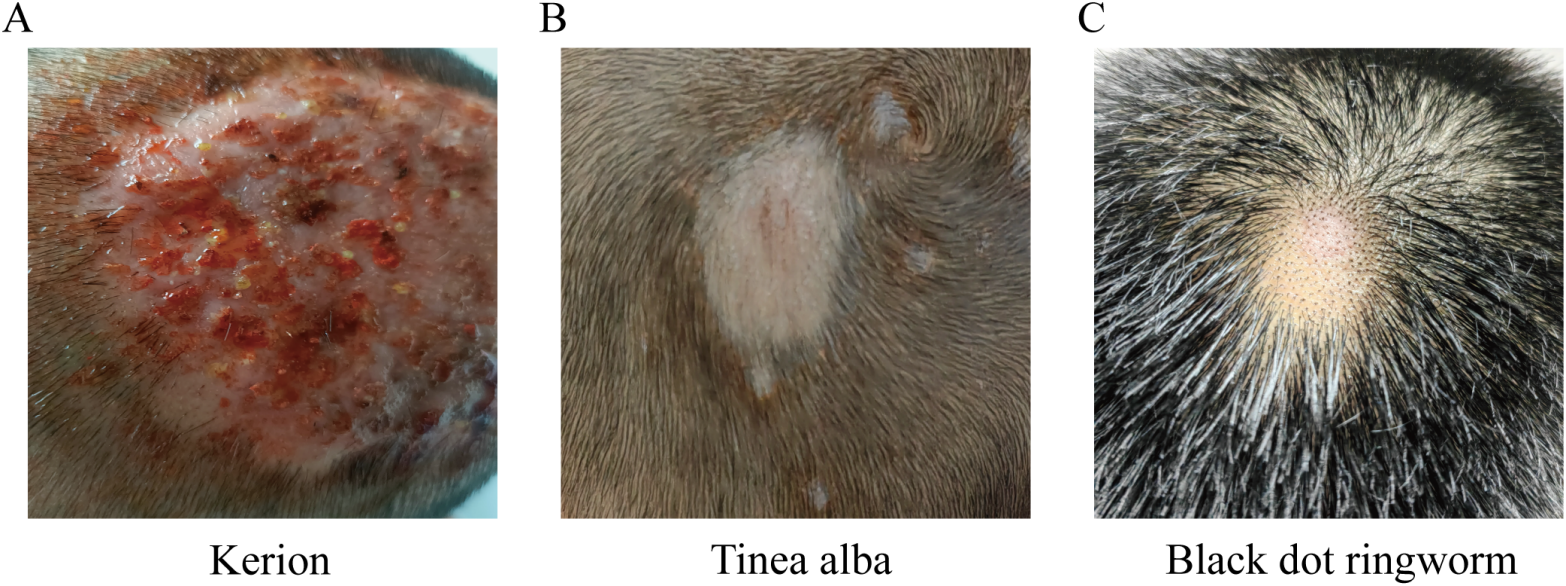
Types of Tinea capitis **(A)** Kerion: one to several round, erythematous, infiltrative, or raised inflammatory plaques with surface clusters of follicular micropustules, honeycomb-like follicular openings, and expressible purulent discharge upon pressure. Hairs in the affected area are loose and easily extracted. **(B)** Tinea alba: Scalp lesions present as gray-white scaly patches, round or oval in shape, with possible satellite lesions. **(C)** Black dot ringworm: Affected hairs break off at the follicular orifice, leaving black, dot-like remnants at the follicular openings.

Diagnosis of tinea capitis was based on typical clinical manifestations, dermoscopy, and positive fungal microscopy and culture. Common types of tinea capitis include kerion (57.89%, 44 cases), tinea alba (35.53%, 27 cases), and black dot ringworm (6.58%, 5 cases). No cases of favus were found. Age group did not significantly affect tinea capitis type (P > 0.05). Most of the patients were children (≤15 years old), and most of the patients had more severe pyophyton (**Figure 1**, **Supplementary Table 1**).

### 3.3 Pathogen composition

**Figure 2:**
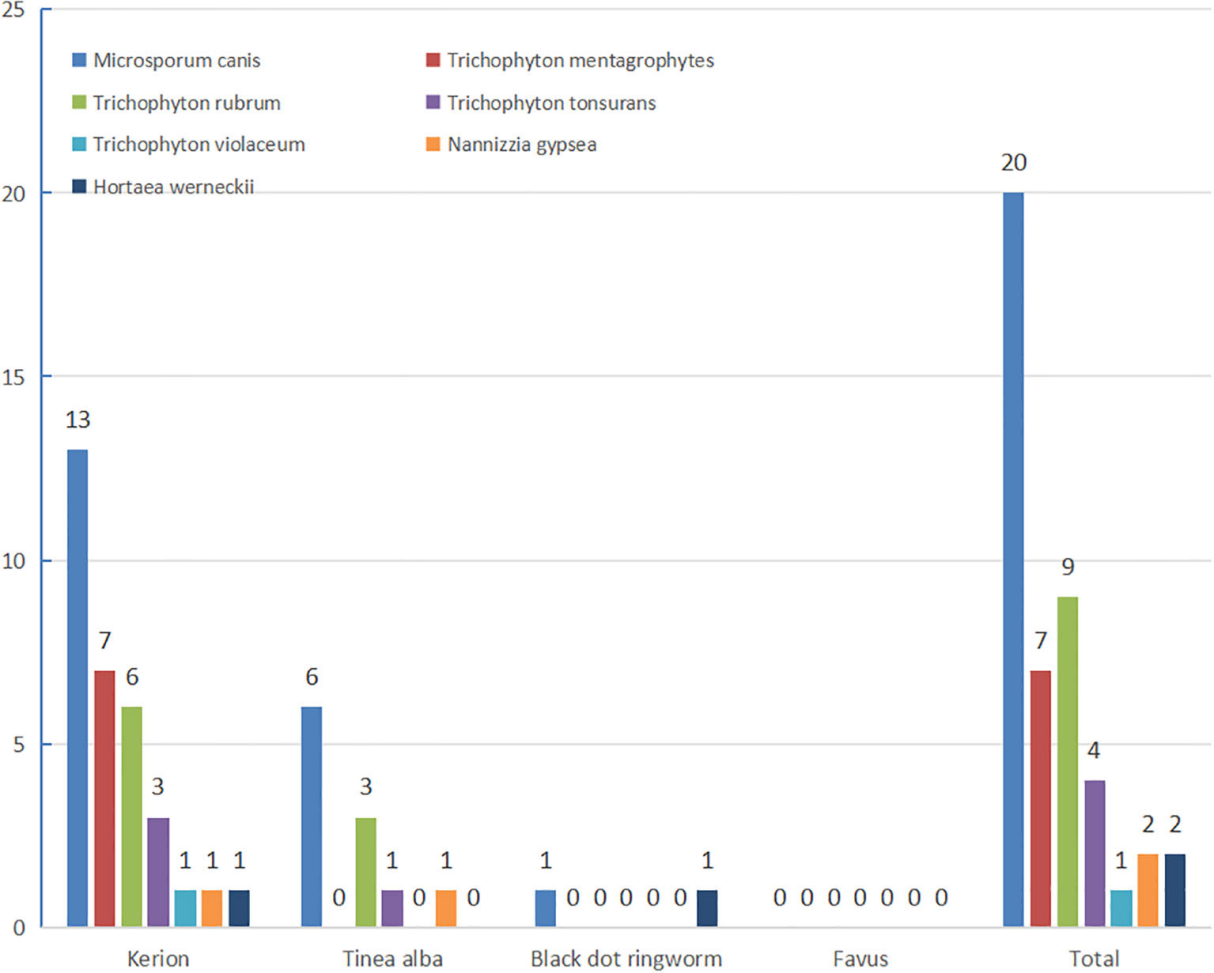
Types and pathogenic bacteria of tinea capitis.

Among 76 clinically diagnosed tinea capitis cases, direct fungal microscopy yielded positive results in 64 cases (84.21% positivity rate). Fungal culture isolation yielded 45 fungal isolates, including 20 presumptive *M*. *canis strains* (later confirmed through rice grain medium phenotyping). All 45 strains underwent morphological identification followed by molecular sequencing. Sequence alignment of the rDNA-ITS region with reference strains in the GenBank database demonstrated ≥99.0% similarity for all isolates. The most positive culture was kerion, followed by tinea alba. The most common pathogenic fungi were zoophilic *M. canis* (43.79%) and anthropophilic *T. rubrum Trichophyton* 9 (19.57%), There were (17.39%) (7 strains). 4 (8.70%) and 1 (2.17%) strains of zoophilic mentagrophytes (*T. mentagrophytes*), anthropophilic Trichophyton tonsurans (*T. tonsurans*) and *Trichophyton violaceum* (*T. violaceum*), respectively. Two strains (4.35%) of *Nannizzia gypsea* were soil ophilic. Two strains of *Hortaea werneckii (H. werneckii*) were also identified. Kerion was predominantly caused by *M. canis* (13 strains) and *Trichophyton mentagrophytes* (7 strains). White tinea (tinea alba) primarily involved *M. canis* (6 strains) and *T. rubrum* (3 strains), while a single case of black dot tinea also demonstrated *M. canis* infection (1 strain) (**Figure 2**).

**Figure 3:**
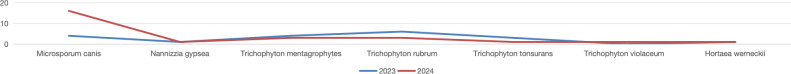
Pathogen composition in 2023 and 2024.

There was no significant difference between tinea capitis type and pathogenic fungi (P>0.05). There was no significant difference in pathogenic fungi detected between 2023 and 2024 (P>0.05). In 2023, mycological analysis identified 19 fungal strains, predominantly *M. canis* (4 strains) and *T. rubrum* (6 strains). In 2024, 26 strains were isolated, with *M. canis* (16 strains) and *Trichophyton mentagrophytes* (3 strains) being the most frequent isolates (**Figure 3**).

### 3.4 Relationship between pathogen species and age groups

*M. canis* was most commonly detected in children, particularly in the 5–10 year-old age group (16 cases, 80%). *M. canis*(20cases), *Nannizzia gypsea*(2cases) and *T. mentagrophytes*(7cases) were only found in children aged 0–10 years.

**Figure 4:**
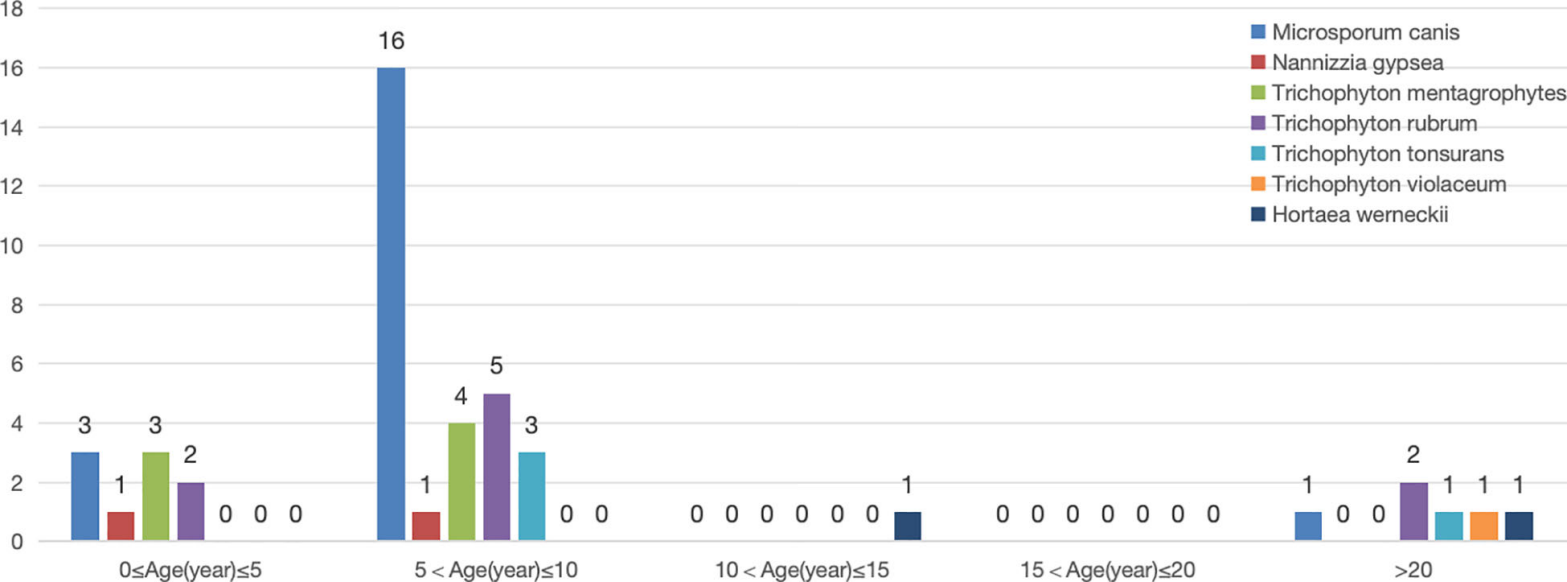
Pathogen species and age groups.

Only 1 strain of *Trichophyton violaceum* was isolated from an adult with tinea capitis. *T. rubrum* (9cases) and *Trichophyton tonsurans* (4cases) were detected in both children and adults. Hortaea werneckii, although not a classic tinea capitis pathogen, was identified in two cases, one from a 13-year-old girl with black dot ringworm and another from a 24-year-old man with kerion. Significant differences were observed in pathogen species distribution across age groups (P < 0.05) (**Figure 4**).

### 3.5 Dermoscopy results

Fifteen patients underwent dermoscopy, with 12 cases of tinea alba, 2 cases of black dot ringworm, and 1 case of kerion. In 12 cases of tinea alba, Morse code-like broken hair, external fungal sheath, black dot sign, spiral hair, and comma or question mark-like hair were observed in 3 (25.00%), 1 (8.33%), 10 (83.33%), 3 (25.00%), and 11 (91.67%) cases, respectively. All five features were present in 3 cases (20.00%). Tinea alba exhibited characteristic dermoscopic features in 91.66% (11/12) of cases. In 2 cases of black dot ringworm, external fungal sheath, black dot sign, and comma or question mark-like hair were observed in 1 (50.00%), 1 (50.00%), and 2 (100%) cases, respectively. One case of kerion showed both external fungal sheath and comma or question mark- like hair (**Supplementary Table 2**).

### 3.6 Treatment and course of tinea capitis

The main treatment for tinea capitis was systemic therapy, primarily oral terbinafine, itraconazole, and griseofulvin. Of the 76 cases, 19 received oral terbinafine, 23 received oral itraconazole, and 5 received both. Two cases used traditional Chinese medicine, and 27 received no oral medication. The main treatment was for severe kerion cases, with 16 cases adding oral prednisone acetate and 25 cases combining antibiotic therapy. Some cases also used photodynamic therapy. The course of treatment ranged from 4 to 8 weeks, with the shortest being 30 days and the longest 180 days.

### 3.7 Follow-up protocol and therapeutic efficacy criteria

Follow-up Protocol, Patients were scheduled for biweekly clinical re-evaluations during active treatment. After clinical resolution was achieved, telephone follow-ups were conducted monthly for two consecutive sessions to monitor recurrence and long-term outcomes.

Therapeutic Efficacy Criteria, Final efficacy assessment was based on clinical manifestations combined with mycological examination (direct microscopy and/or culture) at the end of therapy.

Antifungal discontinuation: Oral medications were discontinued upon confirmation of mycological negativity (negative microscopy/culture). Cure confirmation: Patients underwent periodic re-evaluation post-discontinuation, with cure defined as 2–3 consecutive negative mycological results.

## 4 Discussion

Tinea capitis is an ancient chronic infectious disease. Historical records from the Sui Dynasty (610 AD) mention “White baldness” and “Red baldness”. “White baldness” correspond to modern tinea alba and black dot ringworm. “Red baldness” is the modern favus. Tinea capitis is caused by dermatophyte infections of the scalp and hair, classified into kerion, tinea alba, black dot ringworm, and favus based on the causative agent and host response (Hay, 2017; Jin and Min, 2019).

Children are more susceptible due to incomplete development of sebaceous glands and lack of free fatty acids inhibiting fungal growth (Rui et al., 2017). Children also tend to be less clean and more active, and they regularly engage with animals. In this study, 31 patients (40.79%) had a history of animal contact, with 90.32% being children ≤10 years old and 9.68% adults. Concurrent superficial mycosis at additional sites was present in 12 patients (15.79%).

The incidence ranges from infants to the elderly, but it peaks during preschool and school age. Minors (≤15 years old) accounted for 80.26% of cases, with the majority (62.3%, 38/61) in the 5–10 year-old age group. This aligns with studies from other regions (Zhi et al., 2021; Cheng et al., 2022; Chen and Yu, 2023; Zeng et al., 2023). There were 15 adult cases (19.48%), with a greater percentage of females (male:female ratio = 0.6:1). The adult incidence rate is comparable to studies from Hubei Province but slightly higher than the national average (9.04%), suggesting that adult tinea capitis in Hainan is significant (Liang et al., 2020; He et al., 2021). Adult cases often mimic seborrheic dermatitis or alopecia, leading to potential misdiagnosis. Dermoscopy plays a crucial role in diagnosing tinea capitis. Dermoscopy plays a crucial role in diagnosing tinea capitis (Kumar et al., 2022).

In this study, Dermoscopy was performed in 15 patients, revealing subtle clinical features of tinea alba and black dot tinea in 14 cases. In 91.66% of patients, tinea alba displayed distinctive dermoscopic characteristics.

According to classification criteria, 76 cases were primarily kerion (57.89%), followed by tinea alba (35.53%). This corresponds with international studies but differs from some domestic studies (Zhan et al., 2015; Hamroune et al., 2016; Bassyouni et al., 2017; Wen and Xiao, 2018; Chen et al., 2022; Jing, 2022; Wan and Wu, 2022), presumably due to the higher probability of white ringworm and black dot ringworm to develop as kerion. This, combined with the unique geography and climate of Hainan Island, may explain the higher prevalence of kerion.

Pathogens in Hainan Island children’s tinea capitis from 2023–2024 were predominantly zoophilic *M. canis* and *anthropophilic T. rubrum*. This contrasts with previous studies where *T. mentagrophytes* and *T. tonsurans* were dominant (Farag et al., 2018). This shift aligns with results from southern and northern China = and the 2019–2020 multicenter study (Chen et al., 2022). However, in North Africa (Hamroune et al., 2016; Bassyouni et al., 2017; Farag et al., 2018), East Asia (Lee et al., 2020; Takenaka et al., 2020), Southeast Asia (Tan et al., 2018), and Eastern Europe (Baranová et al., 2018; Colosi et al., 2020), the dominant pathogen is *M. canis*. In South Asia and Western Asia=, *T. violaceum* and *M. audouinii* are predominant, while in the United States, *T. tonsurans* is the most common (Nguyen et al., 2020; Usman et al., 2021).

With economic development and improved hygiene conditions, pet ownership has increased, leading to a rise in zoophilic *M. canis* as a pathogen in tinea capitis. Therefore, preventing and managing pet-associated infections is critical. Notably, two cases of *H. werneckii* were identified, one in a 13-year-old girl with black dot ringworm and another in a 24-year-old man with kerion (Zheng et al., 2024). This halophilic fungus, which causes black dot ringworm, is most commonly seen in tropical and subtropical coastal locations.

Based on predefined therapeutic efficacy criteria, all patients achieved clinical and mycological cure, excluding one pediatric case of Hortaea werneckii-associated tinea nigra lost to follow-up prior to final assessment. Due to inherent limitations in existing diagnostic methods, achieving 100% sensitivity remains elusive for both direct mycological microscopy and fungal culture. Among 76 clinically confirmed tinea capitis cases, direct microscopy detected fungal elements in 64 cases (84.21%), whereas fungal culture confirmed pathogen growth in 45 cases (59.21%). These positivity rates were notably lower than those documented in prior studies (Chen et al., 2022; Zeng et al., 2023), which reported sensitivities exceeding 90% for microscopy and 70% for culture. This discrepancy may be attributed to differences in case numbers across studies, the predominance of kerion (inflammatory tinea capitis) as the clinical subtype, and variations in technical proficiency among laboratory personnel.

In this study, cases were primarily concentrated in Haikou, with sporadic cases in other cities. This limitation might be due to Haikou’s status as a central hub for economics, culture, and transportation, attracting a dense population. Other cities may have fewer cases due to transportation barriers and economic constraints. Additionally, the study focused on dermatology prevention and treatment centers rather than comprehensive hospitals. Limited funding also restricted in-depth investigations in rural areas. Furthermore, no comprehensive fungal testing was conducted on other body parts or animals, limiting the understanding of transmission routes.

## Data Availability

The original contributions presented in the study are included in the article/**Supplementary Material**. Further inquiries can be directed to the corresponding authors.
