# Supplementary material for: Tinea capitis in Hainan: a prospective study
Source: Front Cell Infect Microbiol. 2025 Jun 23;15:1590315. doi: 10.3389/fcimb.2025.1590315 (PMC12229872; doi:10.3389/fcimb.2025.1590315)
Supplement: Supplementary file 1 [file Table1.docx]

Supplementary Material

# Supplementary Tables

For more information on Supplementary Material and for details on the different file types accepted, please see [here](https://www.frontiersin.org/guidelines/author-guidelines#supplementary-material).

## **Supplementary Table 1.** Demographic characteristics and types of tinea capitis in different regions of Hainan Province, 2023-2024

| Regions | Number of cases | | **Sex** | | | **Age groups (years)** | | | | | | | | | | **Types of Tinea capitis** | | | | | | | |
| --- | --- | --- | --- | --- | --- | --- | --- | --- | --- | --- | --- | --- | --- | --- | --- | --- | --- | --- | --- | --- | --- | --- | --- |
|  |  |  | male | | female | 0≤Age≤5 | | 5＜Age≤10 | | 10＜Age≤15 | | 15＜Age≤20 | | Age>20 | | Kerion | | Tinea alba | | Black dot ringworm | | Favus | |
| Haikou City and surrounding areas | 50 | 25 | | 25 | | | 10 | | 26 | | 3 | | 0 | | 11 | | 25 | | 22 | | 3 | | 0 |
| Danzhou City | 2 | 0 | | 2 | | | 0 | | 2 | | 0 | | 0 | | 0 | | 2 | | 0 | | 0 | | 0 |
| Lingao county | 4 | 2 | | 2 | | | 2 | | 1 | | 0 | | 0 | | 1 | | 3 | | 1 | | 0 | | 0 |
| Changjiang City | 3 | 2 | | 1 | | | 0 | | 3 | | 0 | | 0 | | 0 | | 2 | | 1 | | 0 | | 0 |
| Baisha City | 2 | 1 | | 1 | | | 0 | | 1 | | 0 | | 0 | | 1 | | 2 | | 0 | | 0 | | 0 |
| Dongfang City | 4 | 2 | | 2 | | | 1 | | 2 | | 0 | | 0 | | 1 | | 3 | | 1 | | 0 | | 0 |
| Tunchang county | 2 | 2 | | 0 | | | 1 | | 1 | | 0 | | 0 | | 0 | | 1 | | 1 | | 0 | | 0 |
| Qiongzhong county | 1 | 0 | | 1 | | | 1 | | 0 | | 0 | | 0 | | 0 | | 1 | | 0 | | 0 | | 0 |
| Wenchang City | 3 | 2 | | 1 | | | 1 | | 1 | | 0 | | 0 | | 1 | | 2 | | 0 | | 1 | | 0 |
| Wanning City | 3 | 1 | | 2 | | | 1 | | 0 | | 1 | | 0 | | 1 | | 2 | | 1 | | 0 | | 0 |
| Sanya City | 1 | 0 | | 1 | | | 0 | | 0 | | 1 | | 0 | | 0 | | 0 | | 0 | | 1 | | 0 |
| Ledong county | 1 | 0 | | 1 | | | 0 | | 1 | | 0 | | 0 | | 0 | | 1 | | 0 | | 0 | | 0 |
| Total | N=76 | 37 | | 39 | | | 17 | | 38 | | 5 | | 0 | | 16 | | 44 | | 27 | | 5 | | 0 |

## **Supplementary Table 2**. Results of dermoscopy in 15 cases

| Microscopic representation  Types of tinea capitis | Morse code-like broken hair | external fungal sheath | black dot sign | spiral hair | comma or question mark-like hair | Z-shaped hair |
| --- | --- | --- | --- | --- | --- | --- |
| Kerion（n=1) | 0 | 1 | 0 | 0 | 1 | 0 |
| Tinea alba(n=12) | 3 | 3 | 10 | 3 | 11 | 0 |
| Black dot ringworm(n=2) | 0 | 1 | 2 | 0 | 2 | 0 |
| Total(n=15) | 3 | 5 | 12 | 3 | 14 | 0 |
